# Effect of Acid-Stabilizing Hemagglutinin Mutations on Immunogenicity and Heterologous Protection by H1N1 Influenza Virus mRNA-LNP Vaccines

**DOI:** 10.3390/v18040467

**Published:** 2026-04-15

**Authors:** Chet R. Ojha, Samuel W. Rovito, Balaji Banoth, Hyunsuh Kim, Jeremy C. Jones, Mohamad-Gabriel Alameh, Po-Ling Chen, Richard J. Webby, Drew Weissman, Charles J. Russell

**Affiliations:** 1 Department of Host-Microbe Interactions, St. Jude Children’s Research Hospital, Memphis, TN 38105, USAsamuel.rovito@stjude.org (S.W.R.); banoth.balaji@microcrispr.com (B.B.); jeremy.jones@stjude.org (J.C.J.); po-ling.chen@stjude.org (P.-L.C.); richard.webby@stjude.org (R.J.W.); 2 St. Jude Graduate School of Biomedical Sciences, St. Jude Children’s Research Hospital, Memphis, TN 38105, USA; 3 Department of Medicine, University of Pennsylvania, Philadelphia, PA 19104, USAdreww@pennmedicine.upenn.edu (D.W.)

**Keywords:** nucleoside modified mRNA-LNP, influenza vaccines, hemagglutinin, stalk mutations, immunogenicity, heterologous protection

## Abstract

While current influenza vaccines often lack broad protection against antigenically drifted strains, some modified hemagglutinin (HA) protein antigens have shown promise in eliciting broadly neutralizing antibodies against conserved epitopes. During infection, the mildly acidic environment of the late endosome triggers irreversible HA conformational changes resulting in a post-fusion structure with altered antigenicity. While enhancing the stability of other structural class I viral fusion protein antigens has been instrumental in improving the effectiveness of COVID-19 and RSV vaccines, the role of HA stability in influenza vaccine immunogenicity is relatively unclear. Here, we used the nucleoside-modified mRNA-LNP platform to test engineered HA antigens with specific acid-stabilizing mutations (E47K, K58I, R106K, and K153E) in the HA stalk. All mutations increased HA acid stability, but E47K and R106K did not increase immunogenicity. K153E and K58I, but not E47K and R106K, enhanced the cell-surface expression of the HA protein in vitro. In mice, K153E- and K58I-containing mRNA-LNP vaccines elicited increased neutralizing antibody titers against homologous virus. K153E conferred greater protection than wild-type vaccine against lethal heterologous A/PR/8/34 challenge at low doses (0.5–1.0 µg), despite the absence of neutralizing antibodies against the challenge strain. K153E also elicited greater expansion of antigen-specific antibody-secreting cells (ASCs) in the bone marrow, as well as cross-reactive T follicular helper (Tfh) cells in the spleen. For the vaccines studied, increased HA expression was a stronger correlate of mRNA-LNP enhancement than increased HA stability.

## 1. Introduction

Influenza virus infections remain a major public health concern with annual global estimated infection tolls of 1 billion, and mortality of half a million [1,2]. Current seasonal influenza vaccines vary in effectiveness due to rapid antigenic drift of the virus and the mismatch between vaccine strains and circulating variants, often necessitating regular reformulation and annual immunization [3,4]. Consequently, developing a “universal” or broadly protective influenza vaccine that protects against diverse viral subtypes and drifted strains is a critical priority [5,6,7].

Hemagglutinin (HA) is a structural class 1 viral fusion glycoprotein and a key determinant of vaccine-induced immunity, serving as the primary target for neutralizing antibodies. HA is a homotrimer responsible for viral entry through receptor binding and mediation of viral and host–cell membrane fusion [8,9,10]. HA is synthesized as an inactive precursor protein (HA0) and later proteolytically cleaved into a fusion-competent HA1/HA2 complex extracellularly by a trypsin-like protease, or intracellularly by a furin-like enzyme. HA consists of a highly variable, membrane-distal head domain, containing the receptor-binding site and immunodominant epitopes, and a more highly conserved membrane-proximal stalk domain [11,12]. While antibodies against the head are typically strain-specific and sensitive to antigenic drift, antibodies targeting the conserved stalk region can provide broader heterosubtypic protection [13].

Following endocytosis, the acidic environment of the endosome triggers irreversible conformational changes in HA, driving membrane fusion (reviewed in [14]). The pH at which this occurs (activation pH) varies among strains (typically pH 4.8 to 6.2). The conformational stability of HA is a critical factor governing viral infectivity, transmission, and host range. Numerous mutations have been identified that increase the acid stability of the H1N1 HA protein including stalk mutations E47K, K58I, R106K, and K153E (HA2 numbering) [10,14,15,16]. As mutations that stabilize the pre-fusion structures of other class 1 viral fusion glycoproteins have been shown to enhance the display of B cell and T cell epitopes and improve vaccine potency [17,18,19,20,21], we wished to investigate the effects of stabilizing HA mutations on influenza vaccine immunogenicity and protection.

The nucleoside-modified mRNA-lipid nanoparticle (mRNA-LNP) vaccine platform offers a versatile approach that provides opportunities for rapid and easy tailoring of vaccine antigens. mRNA-LNP vaccines also induce potent neutralizing antibody responses and robust T follicular helper (Tfh) cell and germinal center B cell responses, which are essential for durable, high-affinity immunity [13,22,23,24]. However, achieving broad heterologous protection often requires high doses of mRNA or complex vaccination regimens. We hypothesized that combining the potent immunogenicity of the mRNA-LNP platform with HA antigens engineered for increased stability could enhance vaccine properties such as protective breadth and potency, potentially enabling dose sparing.

To study the effect of increased HA stability on influenza vaccine immunogenicity, we generated mRNA-LNP vaccines encoding the full-length HA of A/California/07/2009 (pH1N1), containing either WT or a mutation previously shown to increase HA acid stability (i.e., E47K, K58I, R106K, or K153E). Each mutation increased HA acid-stability but only those that also increased HA cell-surface expression contributed to increased immunogenicity and protection.

## 2. Materials and Methods

### 2.1. Cells and Viruses

Madin-Darby canine kidney (MDCK; CCL-34; ATCC, Manassas, VA, USA) and Vero cells (ATCC CCL-81) cells were maintained in Dulbecco’s Modified Eagle Medium (DMEM, Life Technologies™, Carlsbad, CA, USA). Human embryonic kidney 293T (HEK-293T; ATCC CRL-3216) cells were maintained in Opti-MEM^®^ reduced serum medium (Life Technologies™, Carlsbad, CA, USA). All culturing media were supplemented with 5% HyClone^®^ standard fetal bovine serum (FBS, Life Technologies, Carlsbad, CA, USA) and 1% penicillin/streptomycin (P/S, ThermoFisher Scientific, Waltham, MA, USA) and grown at 37 °C with 5% CO_2_.

H1N1 Viruses A/Brisbane/02/2018, A/California/04/2009 (Cal/09), A/California/07/2009 (CA/09), A/Michigan/45/2015 (MI015), A/Solomon Islands/03/2006 (SI06), A/New Caledonia/20/1999 (NC99), A/Singapore/06/1986 (SG86), A/Chile/01/1983 (CL83) were generously provided by Dr. Ted Ross. H1N1 virus A/Puerto Rico/8/34 (PR8) and H3N2 virus A/Texas/14/2017 (TX017) were generated by reverse genetics and sequence confirmed as described [8].

### 2.2. mRNA Production and LNP Formulation

mRNA-LNP vaccine antigens were produced as described ([13]). Briefly, plasmids of codon-optimized A/Cal/07/2009 (CA09) HA cDNA were synthesized (GenScript, Nanjing, China), and previously identified HA stability mutations E47K, HA2- K58I, HA2-R106K and HA2-K153E (HA-2 numbering) were introduced to generate stable HA variants (GenScript, Nanjing, China). The variant plasmids were then linearized by BSPQI digestion. Modified mRNA transcript synthesis was then performed using the MEGAscript T7 Polymerase kit (ThermoFisher, Waltham, MA, USA), and 1-methylpseudouridine (m1Ψ)-5′-triphosphate (TriLink, San Diego, CA, USA). mRNAs were then capped with cap1 via a ScriptCap m7G capping kit with 2′-O-methyltransferase and polyadenylated to contain 101 nucleotide-long poly(A) tails (CellScript, Madison, WI, USA). Transcripts were then purified by fast protein liquid chromatography, FPLC, using an Akta Purifier (GE Healthcare, Chicago, IL, USA). All mRNAs were analyzed by denaturing or native agarose gel electrophoresis, and stored at −80 °C.

mRNA vaccine transcripts were encapsulated in lipid nanoparticles (LNPs) using a self-assembly process in which an aqueous solution of mRNA at pH 4.0 is rapidly mixed with a solution of lipids dissolved in ethanol. RNA to total lipid ratio was ~0.05 (wt/wt) and the diameter of LNP was ~80 nm as measured by dynamic light scattering using a Zetasizer Nano ZS (Malvern Instruments Ltd., Malvern, UK) instrument. The mRNA-LNP formulations were stored at −80 °C at a concentration of mRNA of ~1 μg/μL.

### 2.3. Western Blot and Flow Cytometry Analysis

HEK-293T cells or mouse bone marrow derived dendritic cells were plated in 12-well plates, then transfected with 1 µg of mRNA-LNP per well. At specified time post-transfection, 5 µg/mL TPCK-treated Trypsin was added for 15 min at 37 °C before cell collection in 1.5 mL Eppendorf tubes. For Western blots, cells were lysed for 30 min, 4 °C with RIPA buffer. Lysates were then resolved by 4 to 12% Bis-Tris gels (ThermoFisher, Waltham, MA, USA), 30 min, 200 V on ice, and protein bands were transferred to polyvinylidene difluoride (PVDF) membranes 90 min, 25 V. The membranes were blocked with 5% (wt/vol) skimmed milk powder in PBST (Phosphate-Buffered Saline [PBS] containing 0.2% Tween 20) for 2 h at room temperature. Next, the membranes were washed with PBST and incubated overnight at 4 °C with anti-influenza A virus HA polyclonal antibody G.618 (BEI resources, Manassas, VA, USA; NR15696) at a 1:2000 dilution. The membranes were washed with PBST and incubated at room temperature for 1 h with anti-rabbit horseradish peroxidase-conjugated secondary antibody. Protein expression was normalized using beta-actin (Abcam, Cambridge, UK; ab8224) as a loading control. Bands were analyzed for mean-gray value using ImageJ v1.54r, where total expression of cleaved HA was defined as HA0 + HA1 + HA2 [8]. Percent HA cleavage was defined as (HA1 + HA2)/(HA0 + HA1 + HA2) × 100.

For flow cytometry, transfected cells were centrifuged, resuspended and washed in fluorescence-activated cell sorting (FACS) buffer (PBS with 1% BSA), and then stained with anti-HA monoclonal antibodies 163-06 (1 µg /sample; kindly provided by Dr. Jarrod Mousa), FluA-20 (Creative Biolabs, Shirley, NY, USA), for 1 h. Samples were then labeled with AlexaFlour 594 goat anti-human IgG secondary antibody (Life Technologies, Carlsbad, CA, USA) for 20 min at 4 °C in the dark. After final wash with FACS buffer, cells were resuspended in FACS + 1 µg/mL 4′,6-Diamidino-2-Phenylindole -DAPI- (ThermoFisher, Waltham, MA, USA) and stored at 4 °C until analysis. The labeled cells were then analyzed by LSRFortessa flow-cytometer and geometric mean fluorescence (GMF) intensities were reported for each variant using FlowJo v10.10.0.

### 2.4. Syncytia Assay to Determine HA Protein pH of Activation Values

The pH of the membrane fusion was measured by syncytia formation assay in mRNA-LNP transfected Vero cells as described previously [8]. Briefly, Vero cells in 12-well plates were transfected with 3 µg of mRNA-LNP vaccine per well. At 16–18 h post-transfection, the cells were washed with PBS and treated with 5 µg/mL TPCK-Trypsin (MilliporeSigma, Burlington, MA, USA) at 37 °C for 15 min. Trypsin-treated cells were subsequently exposed to pH-adjusted PBS^+/+^ (adjusted with 0.1 M citric acid, pH 4.6–6.0) and incubated at 37 °C for 15 min. The cells were then neutralized by the addition of complete growth medium and incubated at 37 °C for 3–4 h prior to staining with the Hema3 Stat Pak per the manufacturer’s instructions (Fisher Scientific, Pittsburgh, PA, USA). Syncytia were visualized by brightfield microscopy and photographed using a Axio Observer (Zeiss, Oberkochen, Germany) inverted microscope with attached digital camera.

### 2.5. Mice Immunization and Influenza Virus Challenge Studies

For mice immunization, mRNA-LNPs were diluted to 1 μg, 0.5 μg or 0.1 μg doses in 50 μL of sterile PBS and kept on ice until injection. Six-week-old female DBA/2J mice (Jackson Laboratory, Bar Harbor, ME, USA) were vaccinated intramuscularly using insulin syringes with 1/2cc 30G ultra-fine needles (BD Biosciences, East Rutherford, NJ, USA). FlucelVax Quadrivalent Inactivated Vaccine (QIV) recommended for 2020 influenza season, and an unrelated mRNA-LNP vaccine (CCR2) were used as control vaccines. Booster doses (same as prime doses) were injected intramuscularly on day 28 post-prime dose in prime-boost experiments. Mouse blood was collected before each immunization on days 0 and 27 from orbital sinus after anesthetizing with isoflurane. Blood was also harvested at day 56 post- prime vaccination for those mice given booster doses. Serum was separated by storing blood overnight at 4 °C, then centrifuging in Eppendorf tubes at 3000 rpm for 15 min. Final storage was at −80 °C. The bone marrow, spleens, and lungs of some mice who received booster doses were then harvested on day 42 post-prime immunization after euthanasia by CO_2_, and stored on ice in PBS + P/S. Mice given a single immunization were challenged intranasally on day 28 post-vaccination with a lethal dose of virus diluted in a total 30 μL volume using PBS. Mice given a booster immunization were intranasally challenged with a lethal dose of virus at day 63 post-prime vaccination (35 days post-booster dose). Cal/09 was administered at a dose of 2 × 10^5^ pfu per mouse for homotypic challenge. 50 TCID_50_ units (5 MLD_50_) PR8 virus per mouse was used as a heterologous challenge virus. Weight loss was monitored for 14 days, and mice losing more than 25% of their initial body weight for two consecutive days were humanly sacrificed. The challenge doses for each virus were determined by dose response studies without vaccination. Mice were randomly assigned to cages by blinded staff, and no animals were excluded from data analyses.

### 2.6. Hemagglutination Inhibition and Neutralization Assays

Mice sera were treated with receptor-destroying enzyme (RDE) to inactivate nonspecific inhibitors by adding 3 parts RDE to one-part sera and incubated overnight at 37 °C. RDE was inactivated by incubation at 56 °C for 30–60 min. Then, 6 parts PBS were added to the RDE-treated sera to make dilution of 1:10. All data was shown as geometric mean + geometric SD.

For hemagglutination inhibition (HAI) titer determination, RDE-treated sera were two-fold serially diluted with PBS in V-bottom microtiter plates. Equal volumes of diluted sera were incubated with indicated influenza virus diluted to 8 hemagglutination units (HAU) at 37 °C for 30 min. Hemagglutination was assessed using Turkey Red Blood Cells (TRBCs, 0.5%). HAI titers were defined as the highest dilution of sera that completely inhibited agglutination of TRBCs.

For neutralization (NT) assays, MDCK cells were plated in 96-well plates and incubated overnight at 37 °C and 5% CO_2_. RDE-treated sera were two-fold serially diluted in a 96-well plate in viral infection medium (DMEM with BSA, vitamins, glutamate, sodium bicarbonate, antibiotic solution). Equal volume of virus (100 TCID_50_ per well) was added to diluted sera and incubated at 37 °C for 1 h. Then, the reactions were added to PBS-washed MDCK cells and incubated at 37 °C 1 h, before replacement with fresh infection media. Hemagglutination was assessed using TRBCs after 72 h of incubation. NT titers were defined as the highest dilution of sera that completely inhibited agglutination.

### 2.7. ELISpots for Antigen-Specific Antibody-Secreting Cells

Mice were vaccinated on days 0 and 28 and sacrificed on day 42 to collect spleen, bone marrow (BM) and lungs. Single cell suspensions from spleen, bone marrow and lungs were prepared by dissociation in GentleMACS C-tubes (Miltenyi Biotec, Bergisch Gladbach, Germany), filtration, and lysis with ammonium–chloride–potassium lysing buffer (Lonza, Basel, Switzerland). The number of antigen-specific antibody-secreting cells was enumerated by ELISpot assay as previously described [25] with slight modifications. Briefly, purified recombinant proteins (CA/09 HA, PR8 HA, and A/Vietnam/1203/2004 H5N1 HA were diluted in PBS to a final concentration of 0.1 μg/mL and 100 μL were applied onto a MultiScreen-IP Filter Plate (Millipore, Billerica, MA, USA). The plate was incubated overnight at 4 °C. After discarding the coating solution and washing, the plate was blocked with RPMI 1640 medium supplemented with 10% FBS for 2 h at 37 °C. Splenocytes and BM cells isolated from immunized mice were treated with ACK (Ammonium–Chloride–Potassium) Lysing Buffer (Gibco™, Burlington, ON, Canada) and added to each well and incubated at 37 °C for 6 h. After incubation, the cells were removed, and the plate was washed with PBS with 0.5% Tween three times. Antigen-specific antibody-secreting cells (ASCs) were detected by anti–mouse IgG-HRP secondary antibody (Southern Biotech, Birmingham, AL, USA). Signals were developed by staining with the chromogen substrate AEC (3-amino-9-ethylcarbazole; Sigma-Aldrich, St. Louis, MO, USA), water was added to stop the reaction. Spots were scanned and counted on an Immunospot analyzer (Cellular Technology Limited, Shaker Heights, OH, USA). All data were shown as mean + SD.

### 2.8. Peptide Stimulation and Intracellular Cytokine Staining of Tfh Cells

Single cell suspension of mouse splenocytes were suspended in RPMI 1640 supplemented with 10% FBS and plated at 5 × 10^5^ cells/well in a 96-well U-bottom plate. The cells were stimulated with 2 μg/mL of peptide stim (A/NY/18/2009 or PR8 HA peptide pool) for 12 h at 37 °C and 5% CO_2_. Golgi plug and Golgi stop (BD Biosciences, East Rutherford, NJ, USA) were added to each well at a concentration of 1:1000 and incubated for an additional 6 h. Samples stimulated with phorbol 12-myristate 13-acetate (PMA) and ionomycin were used as positive controls and matched unstimulated samples for each animal were negative controls [13].

Following stimulation, cells were washed twice and resuspended in 50 μL FACS buffer. Then, the cells were stained with live-dead aqua dead cell stain and a cocktail of fluorescently labeled surface stains (CD3, CD4, CD8, and PD-1) for 30 min at 4 °C (reagents from the St. Jude Flow Cytometry and Cell Sorting Shared Resource). Cells were washed twice with FACS buffer and fixed in Fix/Perm Solution (BD Biosciences) for 20 min at 4 °C. After two washes in Wash/Perm buffer (BD Biosciences), cells were stained for fluorescently labeled intracellular stain cocktail (IFNγ, TNFα, IL21, IL2) for 30 min at 4 °C. Cells were washed twice in Wash/Perm buffer, resuspended in FACS buffer, and analyzed on a FortessaLSR flow cytometer. Data was analyzed using FlowJo v10.10.0 software (BD Biosciences, East Rutherford, NJ, USA). The numbers of activated CD4^+^ and PD1^+^ T cells positive for IL2, IFNγ, IL21, or TNFα after background subtraction for paired unstimulated controls were reported. All data were shown as mean + SD.

### 2.9. Statistical Analyses

Statistical analyses were performed using GraphPad Prism v10.4.0. Significance is shown as * *p* < 0.05, ** *p* < 0.01, *** *p* < 0.001, **** *p* < 0.0001. The significance of all Western blot, cell-surface expression, stalk mAb binding flow cytometry, HAI titer, NT titer, and chimeric HA ELISA data were compared by 2-way ANOVA with Tukey’s multiple comparison correction test. FluA-20 mAb binding over pH was compared to WT by 2-way ANOVA with Dunnett’s multiple comparisons correction test. The pH at which a 50% change in binding was found by fitting data to a 4-point sigmoidal dose response curve via unweighted nonlinear, least-squares regression. Weight loss was compared by matched, mixed effects analysis using the Geisser–Greenhouse variability test, and Tukey’s multiple comparisons correction test. Survival of individual mutants was compared to WT by log-rank (Mantel–Cox) tests. Elispot and intracellular cytokine staining data were compared for significance using ordinary one-way ANOVA with Dunnett’s multiple comparisons correction test.

## 3. Results

### 3.1. Expression of WT and Mutant HA Proteins After mRNA-LNP Transfection In Vitro

**Figure 3:**
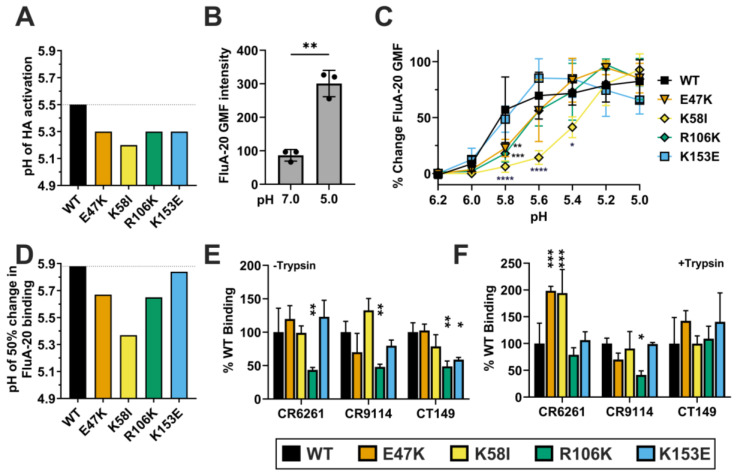
HA stalk mutant stability. (**A**) mRNA-LNPs were transfected into Vero cells, and the pH of activation was determined by syncytia formation assay. (**B**–**D**) pCAGGS expression plasmid HA constructs were transfected into Vero cells and the binding of FluA-20 mAb to was measured by flow cytometry assay. (**B**) FluA-20 binding to WT HA at pH 7.0 and 5.0, with significance determined by Welch’s unpaired *t*-test; data shown as mean ± SD. (**C**) FluA-20 binding to mutants between pH 6.2–5.0, where the percentage of total fluorescence change is plotted as a function of pH and is found in comparison to the total fluorescence change which is defined as maximum GMF—minimum GMF. Data represents *n* = 3 repeat experiments per pH per mutant, shown as mean ± SD. Significance was found by two-way ANOVA with Dunnett’s multiple comparisons correction test. (**D**) The pH of 50% change in FluA-20 binding was defined as the pH of 50% fluorescence change, and found by nonlinear least-squares regression to a four-parameter sigmoidal dose–response curve. (**E**,**F**) Mutant HA binding of broadly neutralizing anti-stalk antibodies (CR6261, CT149 and CR9114) vs. WT as determined by flow cytometry. (**E**,**F**) Mutant HA binding to a panel of mAbs. Data shown as mean + SD. Significance was found by two-way ANOVA with Tukey’s multiple comparisons correction. (**E**) Untreated HA. (**F**) Trypsin-treated HA. For all data: * *p* < 0.05, ** *p* < 0.01, *** *p* < 0.001, **** *p* ≤ 0.0001 indicate statistical significance compared to WT.

**Figure 1:**
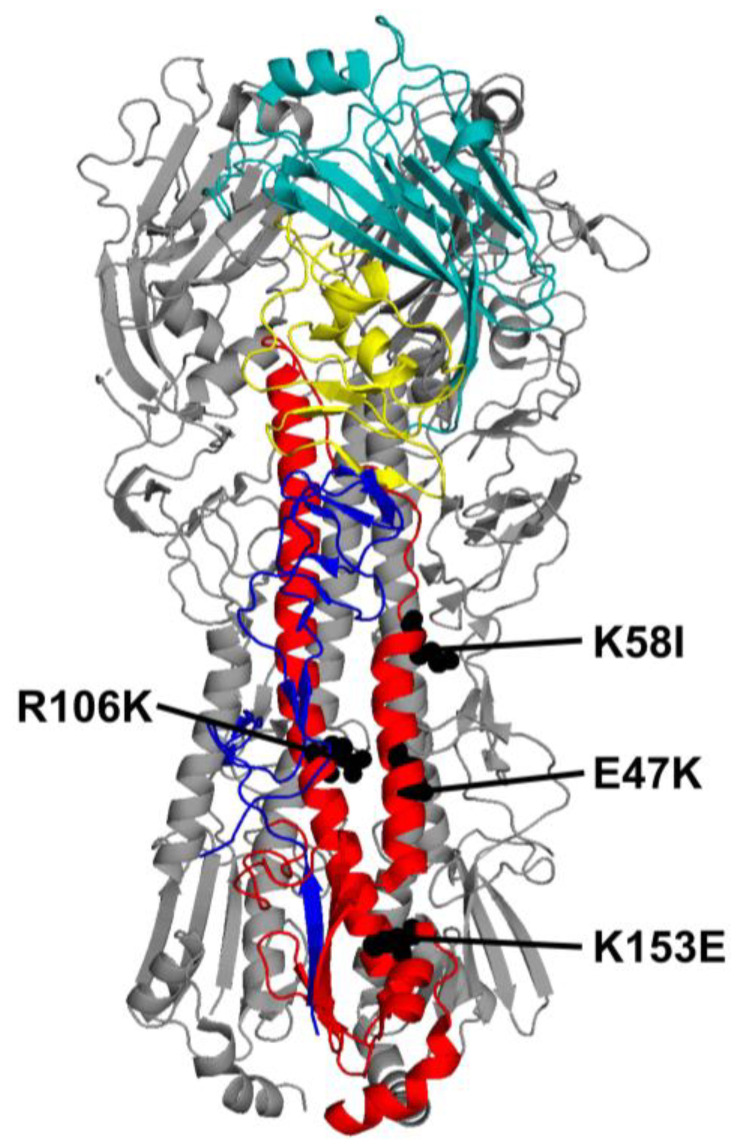
HA protein structure and HA2 stalk mutations. A single HA protomer in the trimer is color coded: HA1 stalk fusion domain (blue), vestigial esterase subdomain (yellow), receptor-binding subdomain (cyan), and HA2 stalk (red). HA2-E47K in helix A introduces an inter-protomeric charge-charge interaction with HA1 E31. HA2-K58I at the C-terminal end of helix A enhances hydrophobic packing into the central triple-stranded coiled coil core. HA2-R106K in the center of the coiled coil reduces electrostatic repulsion at the breakpoint of the HA2 C-helix and D-helix. HA2-K153E at the C-terminal end of the HA2 G-helix introduces a charge-charge interaction with HA2-H26 in the β-turn-β region between the fusion peptide and helix A. HA2-H26 is part of an acid-sensitive histidine switch region (pHS1). A/California/04/2009 (H1N1) PDB structure 3UBE.

**Figure 2:**
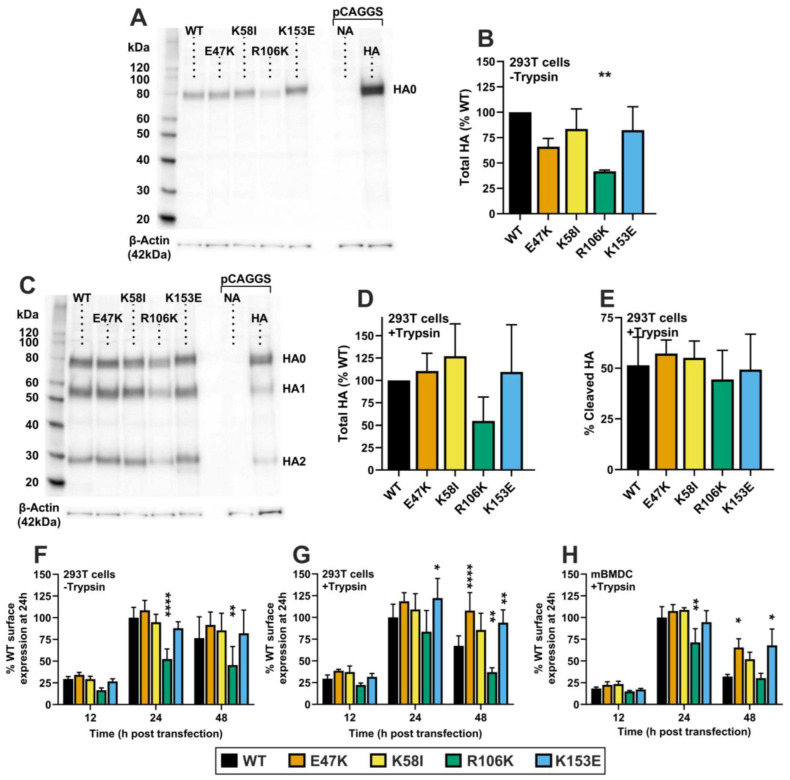
mRNA-LNP vaccine HA antigen expression. (**A**,**B**) Expression of uncleaved HA was measured in HEK-293T cells 24h post-transfection by three independent Western blot experiments. (**A**) Representative blot using pCAGGS-expressed HA and NA as controls. (**B**) HA expression without TPCK trypsin treatment, quantified by mean-gray-values via ImageJ. (**C**–**E**) Expression and percent cleaved HA determined by treating transfected cells with 5 µg/mL TPCK-Trypsin for 15 min, at 37 °C in three separate experiments, analyzing bands by Image J as above. (**C**) Representative blot using pCAGGS controls. (**D**) Total expression calculated as HA0 + HA1 + HA2. (**E**) Proportion of cleaved HA determined by: (HA1 + HA2)/(HA0 + HA1 + HA2) × 100. Cell-surface expression of the HA proteins was measured on transiently transfected HEK-293T cells by flow cytometry (**F**,**G**). (**H**) Cell-surface expression of HA was measured in mouse bone marrow derived dendritic cells transfected with mRNA-LNP *n* = 5. All data are shown as mean ± SD. *p* values were calculated by two-way ANOVA with Tukey’s multiple comparisons correction. * *p* < 0.05, ** *p* < 0.01, **** *p* < 0.0001 indicate statistical significance compared to WT.

The amino-acid sequence of the HA protein from the A/California/07/2009 (CA09) pandemic H1N1 (pH1N1) influenza virus stain was codon-optimized for mRNA expression in the wild-type (WT) mRNA-LNP vaccine. HA2 stalk mutations E47K, K58I, R106K, and K153E were then designed (Figure 1). These mutations arose during the adaptation of pH1N1 viruses to humans or ferrets and were found to decrease the pH required for HA activation and virion inactivation [8,16,26,27,28]. Modified HA mRNA vaccine transcripts were synthesized, then purified by FPLC. mRNAs were then encapsulated into LNPs via laminar-flow mixing and validated for size and uniformity. All data in Figure 2 are shown as mean + SD. Figure 3E,F are likewise shown. Figure 3B,C are shown as mean ± SD.

To study the effects of the mutations on total HA protein expression and cleavage, mRNA-LNPs were transfected into HEK-293T cells and incubated for 24 h before Western blot analysis. In the absence of trypsin, the K58I and K153E mutants showed total expression levels comparable to WT, whereas expression of E47K and R106K were reduced to 66% and 42% of WT levels; *p* = 0.0446, *p* = 0.0016 respectively (Figure 2A,B). Total Expression of TPCK-trypsin-treated HA was not significantly different from WT, though R106K expression trended towards decrease (Figure 2C,D). Cleavage efficiency was consistent across all groups, with approximately 50% of the HA protein cleaved in both WT and mutant transfected cells (Figure 2E).

We further evaluated cell-surface expression kinetics on HEK-293T cells using flow cytometry using the conformation-independent monoclonal antibody (mAb) 163-06, which binds the head region of the pH1N1 HA protein in both pre- and post-fusion conformations. Surface expression of the uncleaved mutants was similar to WT at 24- and 48 h post-transfection, except for R106K, which showed significantly reduced expression of 53% and 61% vs. WT at 24 h and 48 h respectively; *p* < 0.0001, *p* = 0.0010 (Figure 2F). However, when cells were treated with trypsin to mimic the fusion-competent state, surface expression patterns shifted. R106K expression was 85% of WT at 24 h, but only 55% of WT at 48 h; *p* > 0.05, *p* = 0.0015 respectively. As HA0 band intensity was also affected, these results could indicate a decrease in R106K translation. However, K153E showed an increased 122% of WT expression at 24 h, which was maintained as 140% of WT expression by 48 h; *p* = 0.0286, *p* < 0.0064 respectively. E47K expression was also maintained at 140% of WT levels by 48 h post-transfection; *p* < 0.0001 (Figure 2G). This trend was recapitulated in murine bone marrow-derived dendritic cells (mBMDCs), a primary antigen-targeting cell type associated with intramuscular administration of mRNA-LNP vaccines [22]. R106K expression was reduced to 70% of WT at 24 h (*p* = 0.002), while E47K, K58I and K153E expression was maintained at significantly higher levels than WT at 48 h post-transfection, 205%, 173%, and 212%; *p* = 0.0003, *p* = 0.0365, *p* = 0.0001 respectively (Figure 2H).

### 3.2. HA Mutant Stability In Vitro

To confirm the impact of these mutations on acid stability in CA/09 HA, we performed syncytia formation assays in Vero cells. While WT HA mediated cell-to-cell fusion at pH 5.5, all four mutants required stronger acidic conditions to trigger fusion: E47K (pH 5.3), K58I (pH 5.2), R106K (pH 5.3), and K153E (pH 5.3) (Figure 3A). These HA activation pH values after mRNA-LNP transfection were similar to those previously observed during HA expression in virus-infected Vero cells [8,10,28,29].

To further examine the conformational stability of the mutant HA proteins, we measured the binding of mAb FluA-20, which recognizes a trimer interface epitope exposed during low-pH-induced conformational changes [30]. FluA-20 binding to WT HA was higher at pH 5.0 compared to neutral pH 7.0, confirming the exposure of the FluA-20 epitope upon acidification (Figure 3B). Measuring FluA-20 binding to the mutants across a pH range of 6.2 to 5.0 permitted the determination of the pH of 50% change in GMF (Figure 3C,D). E47K, K58I, and R106K binding to FluA-20 was significantly decreased compared to WT at pH 5.8 (*p* = 0.0034, *p* < 0.0001, *p* = 0.0006 respectively). K58I maintained these reductions at both pH 5.6 and 5.4 (*p* < 0.0001, *p* = 0.0115 respectively). These binding profiles were consistent with calculated pH of 50% binding values of pH 5.88 (WT), 5.67 (E47K), 5.37 (K58I), 5.65 (R106K), and 5.84 (K153E) (Figure 3D). Shifts in the acidity needed to stimulate FluA-20 binding to E47K, K58I, and R106K indicate a decrease in epitope accessibility consistent with greater HA head conformational stability. The lack of difference between WT and K153E binding profiles suggests greater functional stability in K153E, without affecting HA head conformation. Collectively, these data indicate that the E47K, K58I, R106K, and K153E mutations enhance HA functional stability but have differing impacts on regional conformation.

Finally, we probed the antigenic conformation of the HA stalk using a panel of broadly neutralizing monoclonal antibodies (bNAbs: CR6261, CR9114, and CT149), which bind to the membrane-proximal region of the HA ectodomain with primary hydrogen-bonded contacts to helix A in HA2 and additional hydrophobic interactions and hydrogen bonds to additional HA1 and HA2 residues in the stalk [31,32,33]. In the absence of trypsin, R106K showed reduced binding to CR6261, CR9114, and CT149, approximately at 44–49% of WT (*p* = 0.0015, *p* = 0.0035, *p* = 0.0041) (Figure 3E). These results are consistent with reduced expression of this mutant as assessed by mAb 163-06 binding to the head region. Interestingly, K153E showed reduced binding to CT149 in its uncleaved state (*p* = 0.0239). However, E47K, K58I, and K153E had similar binding to CR6261 and CR9114. When trypsin-treated, CR9114 binding to R106K was also reduced (*p* = 0.0494). Trypsin cleavage completely restored CT149 binding to K153E. Furthermore, cleavage significantly enhanced the binding of CR6261 to the Helix A (the primary binding region of CR6261) mutants E47K and K58I relative to WT (*p* = 0.0006, *p* = 0.0009 respectively) (Figure 3F). These data suggest that the mutations stabilize the trimer but may also induce minor local conformational alterations that vary based upon proteolytic cleavage.

### 3.3. Immunogenicity and Protection Against pH1N1 Viruses

**Figure 4:**
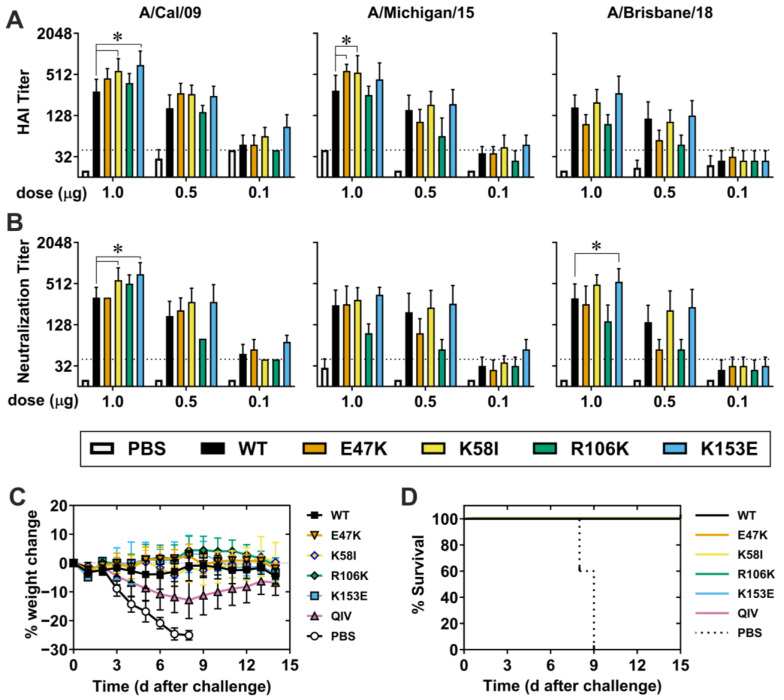
Immunogenicity and protection against pH1N1 viruses. DBA/2 mice (*n* = 5–10) were vaccinated with 0.1, 0.5 or 1 µg mRNA-LNP vaccine. A group of mice was injected only with PBS, and another group was vaccinated with seasonal quadrivalent influenza vaccine (QIV; recommended for 2020) as a control. Peripheral blood was collected 27 days post-vaccination. (**A**) HAI titers, and (**B**) neutralizing titers were measured against a panel of pH1N1 viruses. *p* values for serology were calculated by two-way ANOVA with Tukey’s multiple comparisons with data shown as geometric mean + geometric SD. (**C**,**D**) DBA2/J mice vaccinated with 1 µg of mRNA-LNP were challenged with A/California/07/2009 (pH1N1) on day 28 post-vaccination. Body weight and survival of mice were monitored until day 14 post-challenge. Weight loss is shown as mean ± SD and was compared by matched mixed-effects analysis with a Geisser–Greenhouse variability correction and Tukey’s multiple comparisons correction. Long-rank (Mantel–Cox) tests compared two survival curves. * *p* < 0.05 indicate statistical significance compared to WT mRNA-LNPs.

To evaluate the immunogenicity of a single dose of vaccine, we intramuscularly (i.m.) injected groups of DBA/2J mice with 0.1, 0.5, or 1.0 µg HA mRNA-LNP, collected peripheral blood 27 days later, and measured serum HAI and NT titers against pH1N1 viruses from 2009 (Cal09), 2015 (A/Michigan/45/2015), and 2018 (A/Brisbane/2018). Vaccination with 0.5 or 1.0 µg HA WT mRNA-LNP elicited HAI and NT titers > 1:128 against homologous Cal09 strain, and heterologous A/Michigan/45/2015 and A/Brisbane/2018 strains (Figure 4A,B). Both the K58I and K153E mutants elicited HAI (*p* = 0.0023, *p* < 0.0001) and NT (*p* = 0.0066, *p* < 0.0001) titers higher than WT against Cal09 at the 1.0 µg dose. Similarly, E47K and K58I mutants induced higher titers against MI015 at the 1.0 µg dose (*p* = 0.0424, *p* = 0.0208) but did not impact NT titers. HAI and NT titers against Brisbane/18 were not significantly different than WT except with K153E NT titers reaching significance (*p* = 0.0091). These data suggest that HA-stabilizing mutations K58I and K153E modestly but noticeably increase humoral immunogenicity against matching and closely related viruses.

Separate groups of DBA/2J mice were also vaccinated with 1.0 µg HA mRNA-LNP, quadrivalent influenza vaccine (QIV) control, or PBS (mock control), and all mice were challenged at 28 days with a homologous virus A/California/04/2009. PBS-treated control mice experienced rapid weight loss (>25%) and 100% mortality (Figure 4C,D). QIV-vaccinated mice had an average weight loss of approximately 13%, with 100% survival. The most drastic weight loss among any HA mRNA-LNP-vaccinated mice groups was 2%, with 100%surviving challenge. Overall, a single 1.0 µg dose of HA mRNA-LNP vaccine was highly immunogenic and protective against homologous challenge.

Since K58I and K153E exhibited modest enhancements in immunogenicity against homologous H1N1 strains, we next studied the impact of a booster dose on the immunogenic benefits of these mutants. We primed mice with WT, K58I, and K153E HA mRNA-LNPs, boosted at day 28, and collected blood 56 days after the prime dose. With the prime-boost doses of 0.1 µg, WT and mutant vaccines yielded average HAI and NT titers of at least 960 against pH1N1 strains (Figure S1). At prime-boost dose levels of 1.0 µg and 0.5 µg, K153E significantly increased HAI and NT titers against Cal09 compared to WT; *p* = 0.0048, *p* = 0.0008 respectively, featuring a 2-fold difference at the 0.5 µg dose. This data indicates that the K153E mutant mildly enhanced homologous immunogenicity compared to WT.

### 3.4. Non-Neutralizing Heterologous Immunity Against Seasonal and A/Puerto Rico/8/1934 H1N1 Viruses

**Figure 5:**
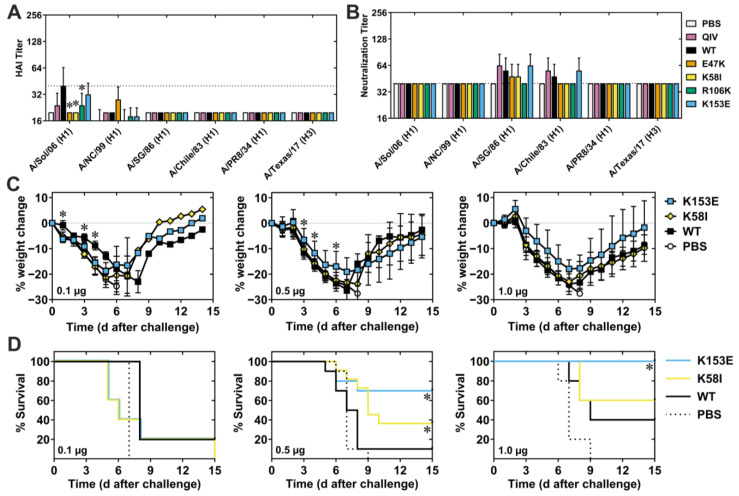
Non-neutralizing heterologous protection enhanced by stalk mutation K153E. DBA2/J mice were intramuscularly vaccinated with 0.1, 0.5 or 1 µg of WT and mutant mRNA-LNP vaccines encoding CA/09 HA. On day 28 after the prime dose, mice were boosted identically as before. Sera was collected on day 56 for analysis against a panel of antigenically distinct seasonal H1N1 viruses as well as A/Texas/14/2017 (H3N2). Significance was found by 2-way ANOVA with Tukey’s multiple comparisons correction, and data are shown as geometric mean + SD. (**A**) HAI titers with dotted line representing seroprotection (titers ≥ 40). The limit of detection was 20, and values below were plotted at 10 for graphing purposes. (**B**) Neutralization titers. The dotted line represents the limit of detection. (**C**,**D**) Mice were then challenged with heterologous virus (PR8) 5 weeks after the boost (63 days after the prime). (**C**) Body weight and (**D**) survival of mice were monitored until day 14 post-challenge. Two independent experiments were performed with *n* = 5 animals in each group for 0.5 µg dose. Weight loss is shown as mean ± SD and was compared by matched mixed-effects analysis using a Geisser–Greenhouse variability correction and Tukey’s multiple comparisons correction. Long-rank (Mantel–Cox) tests were used to compare survival curves. * *p* < 0.05 was considered statistically significant.

To investigate heterologous immunogenicity, we measured HAI and NT titers of sera from prime-boost vaccinated mice using a panel of seasonal H1N1 viruses. HAI and NT titers at 1 µg doses were mostly below the limit of detection for all vaccine groups, though low levels of cross-reactive antibodies were detected against some historical strains (Figure 5A,B). Specifically, E47K, K58I, and R106K HAI titers against A/Solomon Islands/06 were significantly decreased compared to WT while K153E titers were not (*p* < 0.0001). However, both the HAI titers, as well as the differences between them, were small.

We next investigated the breadth of protection against the heterologous H1N1 strain A/PR/8/34 (PR8), which shares approximately 80% sequence identity with Cal09 but is antigenically distinct in the immunodominant head domain [34]. We immunized mice i.m. with 0.1, 0.5, or 1.0 µg vaccine, boosted 28 days later, collected blood 56 days post-prime, and challenged 63 days post-prime with 5 MLD_50_ (50 PFU) of PR8 (Figure 5C,D). Despite the absence of neutralizing antibodies, mice challenged with a lethal dose of PR8 exhibited varying degrees of protection. Mice vaccinated with 0.1 µg K58I and K153E experienced significantly greater weight loss than WT on days 1, 3, and 4, respectively; *p* = 0.0112, *p* = 0.0005, *p* = 0.0023 and *p* = 0.0022, *p* = 0.0417, *p* = 0.0018. However, the peak weight loss and survival rates were similar. At the 0.5 µg dose, K58I and K153E vaccinated mice showed respective 40% and 70% survival compared to 10% survival in the WT group; *p* = 0.0264, *p* = 0.0141. The average weight loss was 26% in the WT, 23% in the K58I, and 19% in the K153E groups. K153E-mice also experienced ameliorated weight loss which reached significance on days 3, 4, and 6; *p* = 0.0379, *p* = 0.0231, *p* = 0.0471 respectively, and trended on days 5 and 7; *p* = 0.0865, *p* = 0.0556. Survival of K153E mice was also pronounced at 1.0 µg, providing 100% protection compared to 40% in WT; *p* = 0.0494 (Figure 5C,D). While not significant, K153E-vaccinated mice did show a slight decrease in weight loss post-challenge, consistent with the survival of these mice. However, 1.0 µg K58I mRNA-LNP provided a 60% survival which was not statistically different than WT (Figure 5C,D). These results indicate that the K153E mutation, at the 0.5 µg and 1.0 µg doses, enhances non-neutralizing responses that lead to increased survival and decreased weight loss against heterologous challenge. Comparatively minor but noticeable increases in the survival of K58I-vaccinated mice at 0.5 µg support a modest protective benefit here as well.

To assess the role of non-neutralizing immunity directed at conserved stalk and cryptic head epitopes, we investigated serum binding activity to a chimeric HA antigen (cH6/1) consisting of an exogenous H6 head domain from A/Mallard/Sweden/81/2002 fused to a H1 Cal/09 stalk domain [35,36]. cH6/1 properly displayed conserved stalk epitopes, as evidenced by strong binding of the stalk-specific bNAb FI6v3 [30,37], while H1-head specific mAbs (5J8 and 2-12C) [38] showed minimal binding (Figure S2A). FluA-20 binding was also observed, indicating the heterosubtypic conservation of the RBD interface epitope in the head region. Analysis of sera collected 28 days post-vaccination revealed that all mice immunized with mRNA-LNP vaccines developed robust levels of stalk-directed IgG antibodies, compared to controls (Figure S2B,C). While the magnitude of the total mutant vaccine stalk-directed IgG response was comparable to WT, the presence of these antibodies across all mRNA-LNP vaccine groups suggests changes in antigen recognition specific to the mRNA-LNP platform. In general, an increased antibody breadth toward the HA stalk was not observed and was considered an unlikely contributor to improved heterologous protection.

### 3.5. Antigen-Specific Antibody-Secreting Cells (ASCs) and T-Follicular Helper (Tfh) Cells

**Figure 6:**
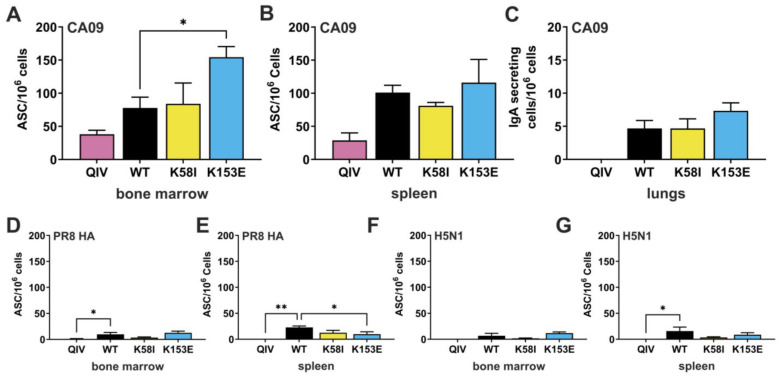
Antigen-specific antibody-secreting cells (ASCs) in bone marrow, spleen, and lungs of vaccinated mice. DBA/2 mice primed on day 0 and boosted on day 28 with 1 µg of mRNA-LNP vaccine were euthanized on day 42 to collect bone marrow, spleen, and lungs. (**A**–**C**) The numbers of antigen-specific ASCs were enumerated by ELISpot assay using purified recombinant HA proteins (A/California/07/2009) coated Multiscreen-IP Filter Plate. (**D**–**G**) ASCs with broader specificity were determined using purified recombinant HA from heterologous strains (A/Puerto Rico/8/1934 or A/Vietnam/1203/2004 H5N1). The spots were scanned and counted on an Immunospot analyzer. One-way ANOVA with Dunnett’s multiple comparisons test was used to compare vaccination groups. The data represent mean + SD of three different mice in each group. * *p* < 0.05, ** *p* < 0.01 indicates statistical significance compared to WT mRNA-LNP vaccine.

To identify the mechanisms contributing to the enhanced immunogenicity and protection by the K153E and K58I mutants, we quantified antigen-specific antibody-secreting cells (ASCs) in lymphoid tissues 42 days post-vaccination (1.0 µg prime-boost). The number of antigen-specific ASCs were quantified by ELIspot assay using purified recombinant HA proteins CA/09, PR8, or A/Vietnam/1203/2004 (H5N1). Compared to QIV vaccination WT mRNA-LNP vaccination induced significantly greater numbers of CA/09-specific and H5N1-specific ASCs in the spleen; *p* = 0.0140, *p* = 0.0358, and greater numbers of PR8-specific ASCs in both the bone marrow and spleen; *p* = 0.0440, *p* = 0.0019 (Figure 6). K153E samples induced approximately two-fold more CA/09-specific ASCs in the bone marrow compared to WT; *p* = 0.0248, but induction was comparable in the spleen (Figure 6A,B). In the lungs, K153E mRNA-LNP induced approximately 55% more CA/09-specific IgA-secreting cells than WT mRNA-LNP, but this difference was not statistically significant; *p* > 0.05 (Figure 6C). While cross-reactive ASCs against PR8 and H5N1 (A/Vietnam/1203/04) were rarer, they were consistently present in mRNA-LNP vaccinated mice but absent in QIV groups (Figure 6D–G). Interestingly, PR8-specific ASCs in the spleens of K153E samples were decreased compared to numbers seen in those of WT samples; *p* = 0.0019. This data contrasts with K153E weight loss and survival against lethal PR8 challenge (Figure 5C,D).

**Figure 7:**
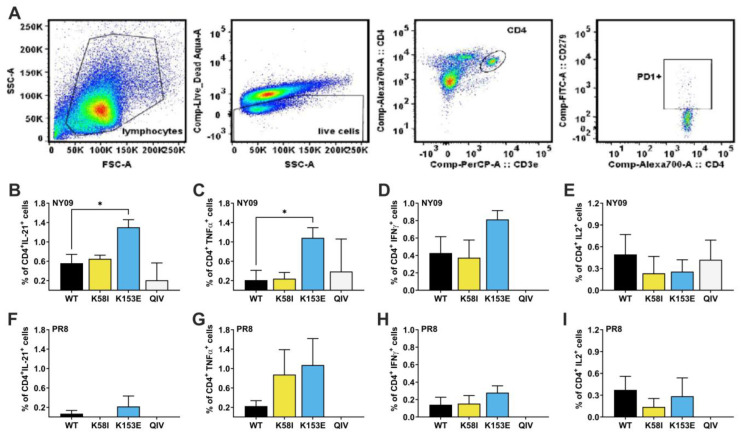
Intracellular cytokines production by antigen-specific T-follicular helper cells (Tfh) in spleen of vaccinated mice. DBA/2 mice were primed at day 0 and boosted on day 28 with 1 µg of mRNA-LNP, and were euthanized on day 42 to collect spleenocytes. The percentage of Tfh cells, CD3^+^CD4^+^CD8^-^PD1^+^ cells positive for IL-21, IFN-γ, TNF-α or IL-2 were determined after background subtraction of paired unstimulated controls. PMA/Ionomycin stimulated and unstimulated match for each sample were included as controls. The data represent mean + SD of three different mice. (**A**) Gating strategy. (**B**–**E**) Respective percentage of Tfh cells positive for IL-21, IFN-γ, TNF-α and IL-2 as detected by multicolor flow cytometry after stimulation with pH1N1 (A/NY/18/2009) or peptide pool. (**F**–**I**) Respective percentage of Tfh cells positive for IL-21, IFN-γ, TNF-α, and IL-2 as detected by flow cytometry after stimulation with A/PR8/34 HA peptide pools. One-way ANOVA with Dunnett’s multiple comparisons test was used to compare vaccination groups; * *p* < 0.05 indicates statistical significance compared to WT mRNA-LNP vaccine.

We next analyzed the breadth of T follicular helper (Tfh) cell recognition and response in the spleen. We isolated and stimulated splenocytes with HA peptide pools of either A/New York/18/2009 pH1N1 (NY09) or PR8. Then, we determined the percentages of antigen-specific CD4^+^PD1^+^ cells producing interleukin-21 (IL21), tumor necrosis factor α (TNFα), interferon γ (IFN-γ), and interleukin-2 (IL2) (Figure 7). The mRNA-LNP vaccines elicited higher frequencies of HA-specific Tfh cells producing IL-21 and IFN-γ compared to QIV. When stimulated with NY/09 peptides, the K153E group showed 2.3-fold higher frequencies of Tfh cells producing IL-21 compared to WT; *p* = 0.012, and a 5.2-fold increase in TNFα^+^ cells compared to WT; *p* = 0.039 (Figure 7B,C). IFN-γ^+^ cells were also present at 1.9-fold increased frequency that trended towards significance; *p* = 0.1009. Furthermore, stimulation with PR8 peptides revealed that both K58I and K153E enhanced the frequency of cross-reactive TNFα^+^ cells by 4.8-fold and 3.9-fold, respectively, compared to WT, though these differences were not statistically significant; *p* > 0.05 (Figure 7G). Likewise, K153E also induced higher percentages of PR8-specific IL21^+^ and IFN-γ^+^ Tfh cells than WT, although this effect was only modest and not statistically significant (*p* > 0.05). These data suggest that K153E facilitates expansion of homotypic T-cell populations, though the contribution of this to heterologous protection is unclear.

Overall, the data showed that introducing HA stabilizing stalk mutation K153E (and to a lesser extent K58I) into an mRNA-LNP vaccine enhanced antigen stability and surface expression and induced increased neutralizing antibody titers against homologous H1N1 virus compared to the wild-type vaccine. These two mutations, especially K153E, also conferred increased protection against a lethal heterologous PR8 challenge despite the absence of detectable neutralizing antibodies. This broadened protection coincided with an expansion of homotypic antigen-specific antibody-secreting cells in the bone marrow and Tfh cells in the spleen.

## 4. Discussion

The relationship between HA stability and immunogenicity is relatively under-studied, with limited literature addressing it in the context of live-attenuated and inactivated vaccines [39,40]. The role of HA stability in the context of mRNA-based vaccines, where the antigen is produced endogenously, is even less defined. In this study, we demonstrate that the pre-fusion conformation stabilizing substitutions K153E, and to a lesser degree K58I, enhance HA mRNA-LNP elicited immunogenicity against antigenically similar HAs as well as the breadth of protection against heterologous challenge. Both mutants feature robust cell-surface expression and functionally stabilized phenotypes that correlate with modest but noticeable increases in homotypic HAI and NT titers. Furthermore, both mutants elicit significantly improved survival against heterologous PR8 challenge at 0.5 µg doses. However, only K153E-vaccinated mice exhibited enhanced ASC and Tfh responses against homotypic and highly related viruses.

Both K58I and K153E stalk mutations lowered the pH of membrane fusion in the context of CA/09 HA, consistent with previous reports [28,41]. However, the pH of conformational change required for FluA-20 binding was reduced in E47K, K58I, and R106K, but similar to WT in K153E (Figure 3). These data suggest that wild-type-like conformational flexibility at the RBD-RBD trimer interface is maintained in K153E while abrogated in the other stalk mutants. This is consistent with the location of K153E being further away from the FluA-20 head epitope than the other mutants. The antigenic impact of this contrast between overall HA stability and regional stability like that of the head remains unclear.

A key finding of this study is the capacity of specific stalk-stabilized HA mRNA vaccines to confer enhanced protective breadth at relatively low vaccine doses. While previous studies reported protection against homologous challenges at doses comparable to those used in this work, protection from heterologous PR8 challenge typically necessitated doses as high as 30 µg [13,42]. Here, both K153E and K58I variants provided survival benefits against PR8 challenge at a dose of just 0.5 µg, with K153E providing complete survivability at 1.0 µg (Figure 5). This protection occurred in the absence of detectable neutralizing antibodies against the challenge virus, implicating protective mechanisms beyond traditional neutralizing head-directed humoral immunity. Indeed, all mRNA-LNP groups elicited robust levels of stalk-reactive IgG compared to QIV, preserving the possibility that specific K153E and K58I local stalk conformations are beneficial to protective breadth for future investigations (Figure S2). The ability to achieve broad protection with reduced mRNA payloads has implications for dose sparing, potentially reducing reactogenicity and manufacturing costs for multivalent formulations.

Mechanistically, the enhanced protection afforded by the K153E mutation could be driven by a superior cellular immune response, particularly by boosting expansion of antigen-specific ASCs and Tfh cells. We observed a significant expansion of pH1N1 HA-specific ASCs in the bone marrow and Tfh cells in the spleen (Figure 6 and Figure 7). Tfh cells are essential for supporting affinity maturation and isotype switching in B cells [23,43,44,45]. Notably, the K153E vaccine induced higher frequencies of Tfh cells producing IL-21 and TNF-α in response to homologous peptides, suggesting that the stabilized antigen may be processed or presented more efficiently to CD4^+^ T cells, or that its prolonged retention promotes a more robust Tfh differentiation and expansion homotypically. However, this effect was not observed heterotypically. It is unclear how HA expression or other factors may contribute to the specific increase in K153E vaccine-elicited T-cell responses seen here. However, the fact that only K153E showed such a phenotype implies an effect specific to this residue. It is possible that induction of cross-reactive CD8^+^ T cells that recognize conserved epitopes (like those in the PR8 peptide pool) may contribute to the heterologous protection observed in K153E. More studies are needed to directly examine the relationship between K153E and T-cell responses.

Structurally, the success of the K153E and K58I mutations highlights the residue-specific nature of HA antigen engineering. K153E is in the HA2 G-helix, where it introduces a salt bridge with H26 in the fusion peptide linker, thereby preventing the premature release of the fusion peptide at low pH [14,28]. K58I, located in HA2 Helix A, enhances hydrophobic packing of the central coiled coil [46,47]. The fact that K153E (a G-helix mutation) outperformed mutations in the central coiled-coil (like R106K) suggests that stabilizing the membrane-proximal region and the fusion peptide niche may be particularly advantageous for maintaining the overall integrity of the trimer on the cell surface without compromising protein folding or transport. In the case of the R106K, which is located at the core of the central coiled coil, reduced HA protein expression may be caused by defects in protein folding or stability during trafficking to the cell surface. Stability-altering substitutions at other structural motifs may also be important in shaping mRNA-LNP immunogenicity. Together these data suggest that the variable impact of HA stabilizing stem mutations on immunogenicity of vaccine may be explained by conformational flexibility associated with individual mutations and local structure. Future studies may address whether the inclusion of multiple stabilizing mutations (for example, K153E and K58I) provides an additive increase in HA expression and immunogenicity, a synergistic effect, or counter-productive effect.

In conclusion, the HA stalk-stabilizing mutation K153E enhanced HA expression and improved mRNA-LNP immunogenicity and protection against heterologous challenge. Not all acid-stabilizing HA stalk substitutions improved mRNA-LNP vaccine-elicited immunity. Specifically, neither E74K nor R106K affected protection against homotypic challenge, with the R106K stabilizing mutant exhibiting modestly reduced homotypic sera reactivity. The K153E mutation had the most noticeable impact, maintaining antigen expression over time, as well as eliciting increases in both humoral and cellular responses. Library-based or rationally designed cell-surface expression screens conducted over time may help to understand the impact of antigen-expression longevity and how it relates to vaccine immunogenicity. Nevertheless, K153E may prove useful in next-generation influenza vaccine development, as it offers enhanced immunogenicity at lower doses against homologous strains, and broadens protection against drifted variants.

## Data Availability

Any additional data will be made available upon written request to the corresponding author.

## Supplementary Materials

The following supporting information can be downloaded at: https://www.mdpi.com/article/10.3390/v18040467/s1, Figure S1: Immunogenicity with prime-boost doses; Figure S2: HA mRNA-LNP vaccine stalk directed serum response in mice [36,48,49].

## Abbreviations

The following abbreviations are used in this manuscript:

ACK	Ammonium–chloride–potassium lysing buffer
ASCs	Antigen-specific antibody-secreting cells
BM	Bone marrow
bNAb	Broadly neutralizing antibody
Brisbane/18	A/Brisbane/02/2018
BSA	Bovine Serum Albumin
CA/09	A/California/07/2009
Cal/09	A/California/04/2009
cDNA	Complementary DNA
cH6/1	Chimeric hemagglutinin containing H6 head and H1 stalk domains
CL83	A/Chile/01/1983
DAPI	4′,6-Diamidino-2-Phenylindole
ELISA	Enzyme-linked immunosorbent assay
ELISpot	Enzyme-linked immunospot
FACS	Fluorescent Activated Cell-Sorting buffer (PBS−/− + 1% BSA)
FBS	Fetal Bovine Serum
FD	Fusion stalk-subdomain of HA1
FPLC	Fast protein liquid chromatography
GMF	Geometric Mean Fluorescence
HA	Hemagglutinin
HAI	Hemagglutination-inhibition
HAU	Hemagglutination units
HEK-293T	Human embryonic kidney 293-transformed cells
HRP	Horseradish peroxidase
IFNγ	Interferon-gamma
IL2	Interleukin 2
IL21	Interleukin 21
i.m.	intramuscular
m1Ψ	1-methylpseudouridine
mAb	Monoclonal antibody
mBMDCs	Murine bone-marrow-derived dendritic cells
MDCK	Madin-Darby canine kidney cells
MI015	A/Michigan/45/2015
MLD_50_	Median Lethal Dose 50%
mRNA-LNP	Nucleoside-modified messenger RNA lipid nanoparticle vaccine
NC99	A/New Caledonia/20/1999
NT	Neutralization assay/titers
NY09	A/New York/18/2009
PBS−/−	Plain Phosphate-buffered saline
PBS+/+	Phosphate-buffered saline containing calcium and magnesium ions
PBST	Phosphate-buffered Saline with 0.2% Tween20 detergent
Pfu	Plaque-forming unit
pH1N1	2009 pandemic-lineage H1N1 viruses
PMA	Phorbol 12-myristate 13-acetate
PR8	A/Puerto Rico/8/1934
PVDF	polyvinylidene difluoride
QIV	Quadrivalent inactivated seasonal influenza vaccine
RBD	Receptor-binding head-subdomain of HA1
RDE	Receptor-destroying enzyme
RIPA	Radioimmunoprecipitation buffer
SG86	A/Singapore/06/1986
SI06	A/Solomon Islands/03/2006
TCID_50_	Tissue Culture Infectious Dose 50%
Tfh	T-follicular helper cells
TNFα	Tumor necrosis factor alpha
TPCK	Tosyl phenylalanyl chloromethyl ketone
TRBCs	Turkey red blood cells
TX017	A/Texas/14/2017(H3N2)
VED	Vestigial-esterase head-subdomain of HA1
WT	Wild-type
