# Supplementary material for: Effect of Acid-Stabilizing Hemagglutinin Mutations on Immunogenicity and Heterologous Protection by H1N1 Influenza Virus mRNA-LNP Vaccines
Source: Viruses. 2026 Apr 15;18(4):467. doi: 10.3390/v18040467 (PMC13119996; doi:10.3390/v18040467)
Supplement: Supplementary file 1 [file viruses-18-00467-s001.zip › viruses-4239569-supplementary.pdf]

Article

Effect of stabilizing hemagglutinin stalk mutations on immunogenicity and heterologous protection by H1N1 influenza virus mRNA-LNP vaccines

Supplementary data

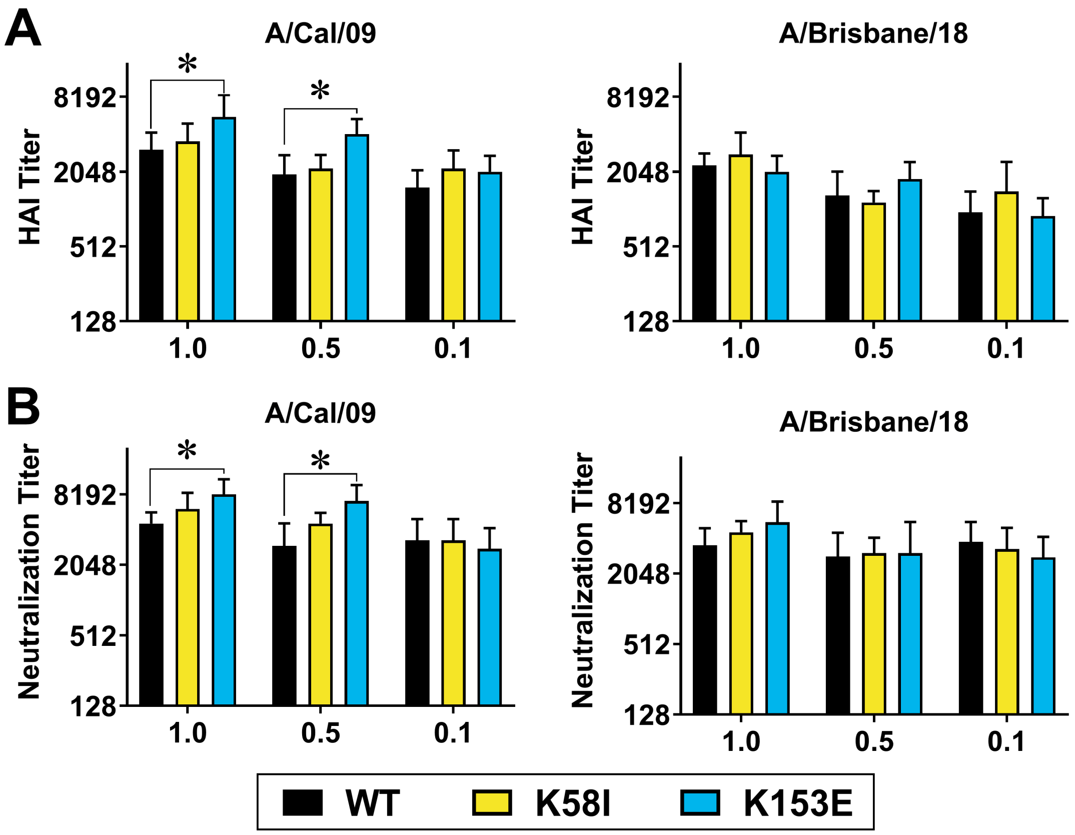

Supplementary Figure S1. Immunogenicity with prime-boost doses. DBA/2J mice (n = 5-10) were vaccinated with 0.1, 0.5 or 1 µg of mRNA-LNP vaccine on day 0 and 28, and blood sera were collected on day 56. (**A**) HAI titers, and (**B**) and neutralization titers were measured against A/Cal/04/2009 and A/Brisbane/02/2018. Data is shown as geometric mean + geometric SD, and *p* values were calculated by two-way ANOVA with Tukey's multiple comparisons correction. $^*p < 0.05$ indicates statistical significance compared to WT mRNA-LNPs.

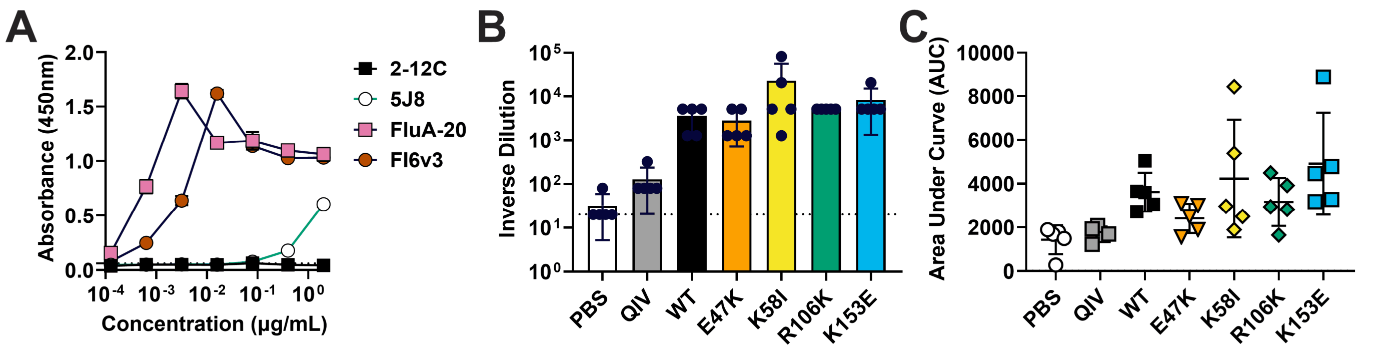

Supplementary Figure S2. HA mRNA-LNP vaccine stalk directed serum response in mice. Chimeric HA antigens with a A/Mallard/Sweden/81/2002(H6N1) head and a A/California/04/2009(H1N1) stalk were prepared as described [1]. (**A**) Monoclonal antibody binding to chimeric HA. 5J8 and 2-12C bind to H1-subtype specific head epitopes [1]. FluA-20 binds to a broadly conserved head epitope [2]. FI6v3 binds to a broadly conserved stalk epitope [3]. mAbs were serially diluted five-fold from a starting concentration of 2 µg/mL. Data represents the mean of n ≥ 3 repeat experiments ± SD. (**B-C**) d28 post-vaccination mouse sera IgG ELISA with chimeric HA. Sera were serially diluted four-fold from a 1:20 starting dilution. Data represents n = 5 mice, four technical replicates per mouse. (**B**) Endpoint titers shown as mean ± SD. (**C**) Mean ± SD of area under the curve. (**B-C**) Results were compared to WT by two-way ANOVA with Dunnett's multiple comparison correction test.

References

1. Alameh, M.G.; Tombacz, I.; Bettini, E.; Lederer, K.; Sittplangkoon, C.; Wilmore, J.R.; Gaudette, B.T.; Soliman, O.Y.; Pine, M.; Hicks, P.; et al. Lipid nanoparticles enhance the efficacy of mRNA and protein subunit vaccines by inducing robust T follicular helper cell and humoral responses. *Immunity* **2021**, *54*, 2877-2892 e2877, doi:10.1016/j.immuni.2021.11.001.
2. Bangaru, S.; Lang, S.; Schotsaert, M.; Vanderven, H.A.; Zhu, X.; Kose, N.; Bombardi, R.; Finn, J.A.; Kent, S.J.; Gilchuk, P. A site of vulnerability on the influenza virus hemagglutinin head domain trimer interface. *Cell* **2019**, *177*, 1136-1152. e1118, doi:10.1016/j.cell.2019.04.011.
3. Corti, D.; Voss, J.; Gamblin, S.J.; Codoni, G.; Macagno, A.; Jarrossay, D.; Vachieri, S.G.; Pinna, D.; Minola, A.; Vanzetta, F. A neutralizing antibody selected from plasma cells that binds to group 1 and group 2 influenza A hemagglutinins. *Science* **2011**, *333*, 850-856, doi:10.1126/science.1205669.
